# Aging With Autism: Racial Disparities in Diagnosis, and Care Among Older Adults With ASD

**DOI:** 10.1093/geroni/igaf122.2094

**Published:** 2025-12-31

**Authors:** Jiameng Yuan, Tianming Guo, Hanzhang Xu

**Affiliations:** Duke University, Durham, North Carolina, United States; University of Rochester, Rochester, New York, United States; Duke University, Durham, North Carolina, United States

## Abstract

Autism Spectrum Disorder (ASD) is a neurodevelopmental condition affecting cognition, psychological well-being, and daily life. As individuals with ASD age, their unique healthcare needs also increased. Racial and ethnic disparities in health and healthcare access have been well-documented, yet little is known about how racial and ethnic disparities affect older adults with ASD. We conducted a scoping review to examine literature on older adults with ASD, focusing on racial and ethnic disparities in diagnosis and healthcare access. We searched PubMed, PsycINFO, and Scopus for peer-reviewed, data-based articles published before December 3, 2024. Two independent raters screened articles based on prespecified inclusion and exclusion criteria and extracted relevant data from each article. Ten eligible articles were included in the final analysis. Compare to White older adults with ASD, Black older adults have higher prevalence of chronic conditions and are less likely to receive diagnoses of their mental health comorbidities. Hispanic older adults with ASD have fewer outpatient mental health visits and are less likely to receive psychotropic medications than White older adults. Older adults with ASD who are Asian/Pacific Islanders were less likely to be dual-eligible for Medicaid and Medicare than their White counterparts across different age groups. Furthermore, ASD is more frequently diagnosed in younger individuals, leading to underdiagnosis in older adults, further exacerbating their healthcare challenges. Recognizing the intersection of aging, autism, and racial disparities is essential for advancing healthcare equity and promoting health aging for older adults with ASD.

